# Vulcanite Litigation

**Published:** 1873-11

**Authors:** Samuel S. White


### ARTICLE III.

### *Vulcanite Litigation.*

Accompanying the November issue of the *Dental Cosmos* is a supplement embodying the answer filed in the Pennsylvania cases, in which are set up, in a form responsive to the last edition of the bills of complaint filed by the Goodyear Dental Vulcanite Company, all the defenses which the counsel in those cases have raised. This answer is printed with blanks for the title of the cause, and name of defendant, etc., thus making it immediately available wherever the *Dental Cosmos* reaches.

The variations in minor points of practice in different sections of the country render it advisable that local counsel should be employed in each case, to see that all matters of form are duly observed. Competent attorneys can be found at every point, and we know of nothing better that can be done than the submission to such counsel of the answer above referred to, as affording by very simple adaptation, a ready means, after appearance is entered, of complying seasonably with the next requirement in equity, (in other words for filing the answer,) and thus avoiding what has heretofore been so fruitful a source of disaster in these suits, to wit: *decrees pro confesso for want of an answer.*

This is intended as a comprehensive and pertinent response to the very large number of letters constantly received, inquiring as to the status of the Cummings Patent, and the progress of suits under it designed to test its validity, but to which it is impossible, by reason of their number, to send individual replies.

To omit nothing that is likely to save the dentist from embarrassment, a memorandum is appended of the order of these proceedings, in case any action should be necessary before counsel can be reached.

First, every suit in equity must be commenced by filing a bill in the office of the clerk of the United States Circuit Court, within and for the district in which the defendant resides, or is found at the time he is served with the process (subpnœa). When such a bill is so filed, the clerk of the court issues a subpœna, which is in the following form:

UNITED STATES, } *sct.*

.................... DISTRICT OF ....................

THE PRESIDENT OF THE UNITED STATES.

*To* ..........................................................................

For certain causes offered before the Circuit Court of the United States in and for the District of , in the Circuit, WE COMMAND and strictly enjoin you, that, laying all other matters aside, and notwithstanding any excuse, you personally be and appear before the JUDGES OF THE SAID COURT, at a Session of the Same Court, to be holden at , on the first Monday of next, to answer concerning those things which shall then and there be objected against you, and to do further and receive what the said Court shall have considered in this behalf. And have you then there this writ. And this you are in nowise to omit, under the penalty of FOUR HUNDRED DOLLARS.

---

**NOTE.—The Defendant in this case required to enter appearance in the Clerk's Office of said Court, on or before the first Monday of next, otherwise the Bill may be taken *pro confesso.***

[A TRUE COPY.]

Witness the Honorable
Chief Justice of the Supreme Court of the United States,
at , this day of
1873, and in the ninety- year of the Independence
of the said United States.

..................................................
*U. S. Marshal,*

..................................................

*Clerk of Circuit Court, U. S.*

Until the service of this subpœna by a United States marshal or his deputy, the defendant named in the bill filed need not pay any attention to the suit; but when so served with such paper, the defendant should immediately consult counsel and see that his appearance is entered according to the requirements of the subpnœa, and, in due course, that his answer is filed. The answer must in all cases be filed on or before the first Monday of the next month after the appearance is entered, without any other notice than the Subpnœa itself; the United States Court practice differing in this respect from the Usual State Court practice, which does not require an answer without notice or rule. From this point the proceedings vary according to the views of the complainants; and as it is impossible to anticipate which one of the several courses open to them they will adopt, counsel should be consulted whenever any further step is taken.

This answer was originally filed in Pennsylvania, New Jersey, and Delaware suits, and also in a suit in Massachusetts brought against Dr. Daniel H. Smith, of Holyoke, who employed counsel at Boston. The result has been an agreement between the counsel in all the above suits to complete and try the Massachusetts suit as a test case, meanwhile suspending all proceedings in the Pennsylvania, New Jersey, and Delaware suits, involving the same defense. The test case will probably be tried in December or January next, and the decision in that case will determine the others which depend upon it, leaving for either side an appeal to the Supreme Court of the United States to settle finally the questions raised in this answer as to the invalidity of the Cummings Patent. Under the arrangement above stated the Company have put in their *prima facia* case, and the defendant is to put in his proofs by the first of November next. Having thus explained the present and prospective situation, we have only to add: Let every dentist WAIT AND WATCH.

SAMUEL S. WHITE.

OCTOBER, 1873.

---

